# Low plasma lysophosphatidylcholines are associated with impaired mitochondrial oxidative capacity in adults in the Baltimore Longitudinal Study of Aging

**DOI:** 10.1111/acel.12915

**Published:** 2019-02-04

**Authors:** Richard D. Semba, Pingbo Zhang, Fatemeh Adelnia, Kai Sun, Marta Gonzalez‐Freire, Norman Salem, Nicholas Brennan, Richard G. Spencer, Kenneth Fishbein, Mohammed Khadeer, Michelle Shardell, Ruin Moaddel, Luigi Ferrucci

**Affiliations:** ^1^ Wilmer Eye Institute Johns Hopkins University School of Medicine Baltimore Maryland; ^2^ National Institute on Aging, National Institutes of Health Baltimore Maryland; ^3^ DSM Nutritional Products Columbia Maryland

**Keywords:** aging, lysophosphatidylcholine, metabolomics, mitochondria, skeletal muscle

## Abstract

The decrease in skeletal muscle mitochondrial oxidative capacity with age adversely affects muscle strength and physical performance. Factors that are associated with this decrease have not been well characterized. Low plasma lysophosphatidylcholines (LPC), a major class of systemic bioactive lipids, are predictive of aging phenotypes such as cognitive impairment and decline of gait speed in older adults. Therefore, we tested the hypothesis that low plasma LPC are associated with impaired skeletal muscle mitochondrial oxidative capacity. Skeletal muscle mitochondrial oxidative capacity was measured using in vivo phosphorus magnetic resonance spectroscopy (^31^P‐MRS) in 385 participants (256 women, 129 men), aged 24–97 years (mean 72.5) in the Baltimore Longitudinal Study of Aging. Postexercise recovery rate of phosphocreatine (PCr), *k*_PCr_, was used as a biomarker of mitochondrial oxidative capacity. Plasma LPC were measured using liquid chromatography–tandem mass spectrometry. Adults in the highest quartile of *k*_PCr_ had higher plasma LPC 16:0 (*p* = 0.04), 16:1 (*p* = 0.004), 17:0 (*p* = 0.01), 18:1 (*p* = 0.0002), 18:2 (*p* = 0.002), and 20:3 (*p* = 0.0007), but not 18:0 (*p* = 0.07), 20:4 (*p* = 0.09) compared with those in the lower three quartiles in multivariable linear regression models adjusting for age, sex, and height. Multiple machine‐learning algorithms showed an area under the receiver operating characteristic curve of 0.638 (95% confidence interval, 0.554, 0.723) comparing six LPC in adults in the lower three quartiles of *k*_PCr_ with the highest quartile. Low plasma LPC are associated with impaired mitochondrial oxidative capacity in adults.

## 1 INTRODUCTION

Progressive mitochondrial dysfunction is an important hallmark of aging (López‐Otín, Blasco, Partridge, Serrano, & Kroemer, 2013). A variety of changes in mitochondria have been described in aging skeletal muscle, including a reduction of number, morphological changes, reduced oxidative phosphorylation efficiency that impairs ATP production, and excess release of reactive oxygen species that cause oxidative damage and possibly accumulation of mitochondrial DNA mutations (Gonzalez‐Freire et al., 2015; Kent & Fitzgerald, 2016). The decline of skeletal muscle mitochondrial oxidative capacity has been associated with lower gait speed, especially in task that require endurance (Choi et al., 2016). Higher physical activity has been associated with higher mitochondrial mass and function (Kent & Fitzgerald, 2016). Insulin resistance is associated with lower mitochondrial function, although the direction of this association is still a matter of discussion (Fabbri et al., 2017).

Recent epidemiological studies show that low concentrations of circulating species in the lipid class of lysophosphatidylcholines (LPC) are independent predictors of several aging phenotypes. Low plasma LPC 18:2 predicted memory impairment and/or Alzheimer's disease in a case–control study (Mapstone et al., 2014). Low serum LPC 17:0 and 18:2 predicted incident myocardial infarction in population‐based studies (Ward‐Caviness et al., 2017). In community‐dwelling adults ≥50 years, low plasma 18:2 predicted greater decline of gait speed (Gonzalez‐Freire et al., 2015). LPC are biologically active lipids that comprise a major class of lipids in human plasma. LPC can serve as ligands for specific G protein‐coupled signaling receptors and as a precursor to lysophosphatidic acid (LPA). LPA is an intermediate in the biosynthetic pathway of cardiolipin, an important dimeric phospholipid that is found almost exclusively in the inner mitochondrial membrane (Schlame & Greenberg, 2017). LPA also has specific receptors involved in growth and differentiation (Aikawa, Hashimoto, Kano, & Aoki, 2015).

We hypothesized that lower plasma LPC were associated with lower mitochondrial oxidative capacity. To address this hypothesis, we measured plasma LPC in a sample of adults who had muscle bioenergetics assessed using phosphorus magnetic resonance spectroscopy (^31^P‐MRS) for quantifying postexercise recovery rate of phosphocreatine (PCr), *k*_PCr_, a measure of mitochondrial oxidative capacity.

## 2 RESULTS

**Figure 1:**
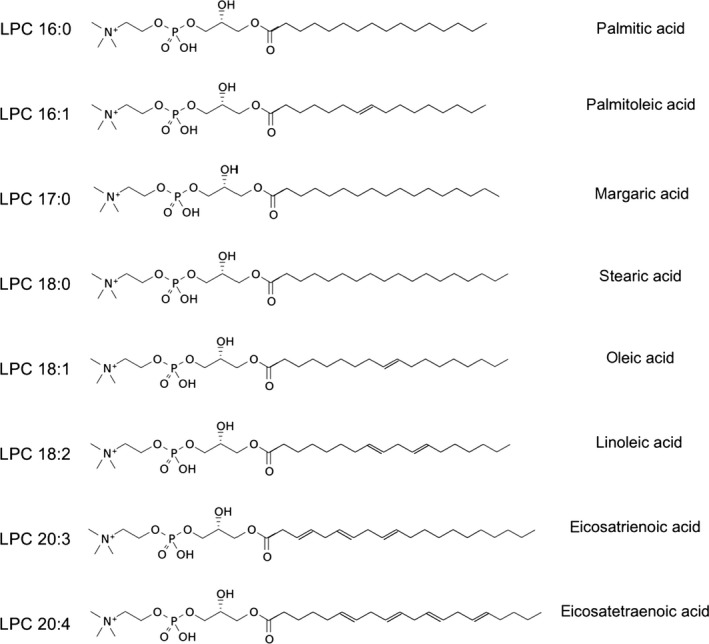
Chemical structure of lysophosphatidylcholine molecular species with fatty acid group noted on the right column. LPCs consist of a glycerol backbone, a phosphate head group at the sn‐3 position, a choline (C_5_H_14_NO), and a single fatty acid chain at the sn‐1 or sn‐2 position. The LPC shown have fatty acids that vary from 16 to 20 carbons in length with the number of double bonds ranging from 0 to 4. The sn‐1 isomer is shown here for simplicity. LPC 20:3 and 20:4 isomers are not distinguished by this LC‐MS/MS assay

The characteristics of the study population by quartile of mitochondrial oxidative capacity (*k*_Pcr_) are shown in Table 1. The mean (standard deviation) for kPCr was 0.020 (0.005). Adults with higher *k*_Pcr_ were significantly younger and taller and more likely to be male. There were no significant differences across quartiles of *k*_PCr_ by weight or BMI. Aerobic capacity (VO_2_ max) was significantly higher in adults with higher *k*_PCr_. Eight LPC species were measured using liquid chromatography–tandem mass spectrometry (LC‐MS/MS). The chemical structures and fatty acid chains of the LPC species are shown in Figure 1. Mean plasma LPC concentrations by quartile of mitochondrial oxidative capacity are shown in Table 2. LPC 16:1, 17:0, 18:0, 18:1, 18:2, and 20:3 concentrations were significantly higher across quartiles of *k*_PCr_, adjusting for age, sex, and height. Adults in the highest quartile of mitochondrial oxidative capacity were also compared with the lower three quartiles combined (Table 3). Higher plasma LPC 16:0, 16:1, 17:0, 18:1, 18:2, and 20:3 concentrations were significantly associated with higher *k*_PCr_ in multivariable linear regression model adjusting for age, sex, and height (*p* < 0.05). LPC 18:0 and 20:4 were higher in the highest quartile of mitochondrial oxidative capacity compared with the lower three quartiles in the models above, although differences were not statistically significant (*p* = 0.07, *p* = 0.09, respectively). In an alternative multivariate linear regression model adjusting for age and sex but not height, higher plasma LPC 16:1 (*p* = 0.005), 18:1 (*p* = 0.001), 18:2 (*p* = 0.008), and 20:3 (*p* = 0.001) were associated with higher *k*_PCr,_but plasma LPC 16:0, 17:0, 18:0, and 20:4 were not significantly associated with *k*_PCr._ Additional analyses were conducted using an interaction term between *k*_PCr_ and sex added to the multivariable linear regression model described above to determine whether the relationship detected was between men and women. The *p*‐values for the interaction term (*k*_PCr_ and sex) for the six different LPC species ranged between 0.19 and 0.98.

**Table 1:** Characteristics of the 385 study participants, aged 24–97 years, by quartile of mitochondrial oxidative capacity (*k*_PCr_)

Characteristic^a^	Quartile 1 <0.0168 (*n* = 96)	Quartile 2 0.0168–0.0195 (*n* = 96)	Quartile 3 0.0196–0.0232 (*n* = 97)	Quartile 4 >0.0232 (*n* = 96)	*p* b
Age, years	76.9 (11.2)	75.2 (10.5)	70.4 (13.8)	67.7 (13.5)	<0.0001
Female sex, %	57.6	65.1	50.0	45.4	0.03
Height, cm	165 (9)	165 (9)	168 (10)	168 (8)	0.003
Weight, kg	73.6 (16.5)	73.5 (14.5)	77.9 (16.3)	75.1 (15.1)	0.09
BMI, kg/m^2^	26.9 (4.4)	26.8 (3.9)	27.3 (4.5)	26.2 (4.1)	0.19
VO_2_ max	18.9 (4.7)	19.3 (4.3)	22.0 (6.2)	25.6 (6.8)	<0.0001

a Mean (*SD*).

b Wilcoxon rank‐sum tests for continuous variables; Pearson chi‐square test for categorical variables.

**Table 2:** Log plasma lysophosphatidylcholine (LPC) molecular species concentrations by quartile of mitochondrial oxidative capacity (*k*_PCr_) adjusted by age, sex, and height

LPC species^a^	Quartile 1 <0.0168 (*n* = 96)	Quartile 2 0.0168–0.0195 (*n* = 96)	Quartile 3 0.0196–0.0232 (*n* = 97)	Quartile 4 >0.0232 (*n* = 96)	*p* b
LPC 16:0	4.81 (4.75, 4.87)	4.83 (4.78, 4.89)	4.82 (4.77, 4.88)	4.89 (4.83, 4.95)	0.20
LPC 16:1	1.11 (1.04, 1.18)	1.17 (1.10, 1.24)	1.21 (1.14, 1.28)	1.28 (1.22, 1.35)	<0.0001
LPC 17:0	0.82 (0.75, 0.88)	0.85 (0.78, 0.91)	0.83 (0.76, 0.89)	0.92 (0.86, 0.99)	0.03
LPC 18:0	3.64 (3.59, 3.70)	3.67 (3.61, 3.72)	3.64 (3.58, 3.70)	3.71 (3.65, 3.77)	0.004
LPC 18:1	3.15 (3.09, 3.22)	3.19 (3.12, 3.25)	3.21 (3.15, 3.28)	3.33 (3.27, 3.40)	0.0002
LPC 18:2	3.63 (3.55, 3.70)	3.67 (3.59, 3.74)	3.71 (3.64, 3.78)	3.81 (3.73, 3.88)	0.01
LPC 20:3	0.75 (0.66, 0.83)	0.78 (0.70, 0.86)	0.88 (0.80, 0.96)	0.98 (0.90, 1.07)	0.0005
LPC 20:4	2.23 (2.14, 2.31)	2.14 (2.06, 2.23)	2.13 (2.04, 2.21)	2.25 (1.16, 2.34)	0.08

a Least squares mean (95% confidence interval), comparison across all four quartiles.

b Multivariable linear regression.

**Table 3:** Multivariable linear regression model of log plasma lysophosphatidylcholines (LPC) and mitochondrial oxidative capacity (*k*_PCr_)^a^

LPC species	Model adjusted for age, sex, and height		
	*β*	*SE*	*p*
LPC 16:0	0.07	0.03	0.04
LPC 16:1	0.11	0.04	0.004
LPC 17:0	0.09	0.04	0.01
LPC 18:0	0.06	0.03	0.07
LPC 18:1	0.14	0.04	0.0002
LPC 18:2	0.14	0.04	0.002
LPC 20:3	0.17	0.05	0.0007
LPC 20:4	0.09	0.05	0.09

a Comparing adults in top quartile versus the lower three quartiles of *k*_PCr_.

**Figure 2:**
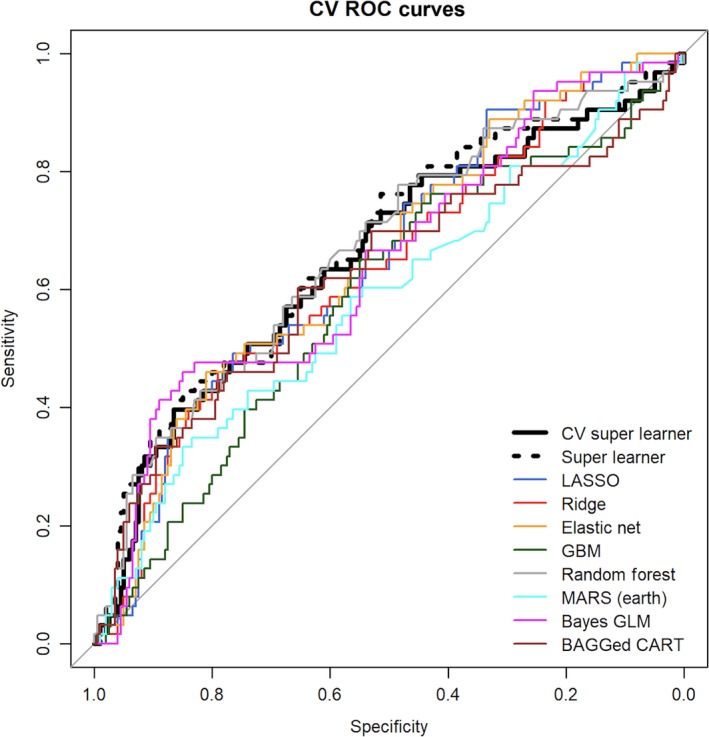
Cross‐validated receiver operating curves of the relationship of the 6 significant plasma LPC species with the highest quartile of *k*_PCr_ using Super Learner, cross‐validated (CV) Super Learner, and eight machine‐learning algorithms. BAGGed CART, bootstrap aggregated classification and regression tree; CV, cross‐validated; LASSO, least absolute shrinkage and selection operator; GBM, generalized boosted regression; GLM, generalized linear model (i.e., main effects logistic regression); MARS, multivariate adaptive regression splines

Multiple machine‐learning classification algorithms were combined using the SuperLearner algorithm, described in Methods, to assess the ability of the six LPC (16:0, 16:1, 17:0, 18:1, 18:2, 20:3) associated with *k*_PCr_ to classify those in the highest quartile of *k*_PCr_ (Semba et al., 2016; van der Laan, Polley, & Hubbard, 2007). Our objective was to report a single global measure describing the strength of association between the multiple LPCs and *k*_PCr_. Cross‐validated receiver operating characteristic curves (ROC) of the relationship of the six LPC with* k*_PCr_are shown in Figure 2. The cross‐validated Super Learner cross‐validated area under the ROC curve was 0.638 (95% Confidence Interval 0.554, 0.723) comparing adults in the lower three quartiles of* k*_PCr_ with those in the highest quartile. An additional machine‐learning analysis using the SuperLearner algorithm was conducted which included the three most significant LPC species (16:1, 18:1, 20:3). In this additional model, cross‐validated Super Learner cross‐validated area under the ROC curve was 0.640 (95% Confidence Interval 0.562, 0.719) comparing adults in the lower three quartiles of k_PCr_ with those in the highest quartile.

## 3 DISCUSSION

The present study shows that higher plasma LPC with fatty acid chains 16:0 (palmitic acid), 16:1 (palmitoleic acid), 17:0 (margaric acid), 18:1 (oleic acid), 18:2 (linoleic acid), and 20:3 (eicosatrienoic acid, n‐3, dihomo‐gamma‐linolenic acid, n‐6, and/or mead acid, n‐9) are associated with higher mitochondrial oxidative capacity in adults. The major species of 20:3 and 20:4 are in the n‐6 configuration in plasma. To our knowledge, this is the first study to identify circulating biomarkers associated with mitochondrial oxidative capacity in skeletal muscle in humans.

**Figure 3:**
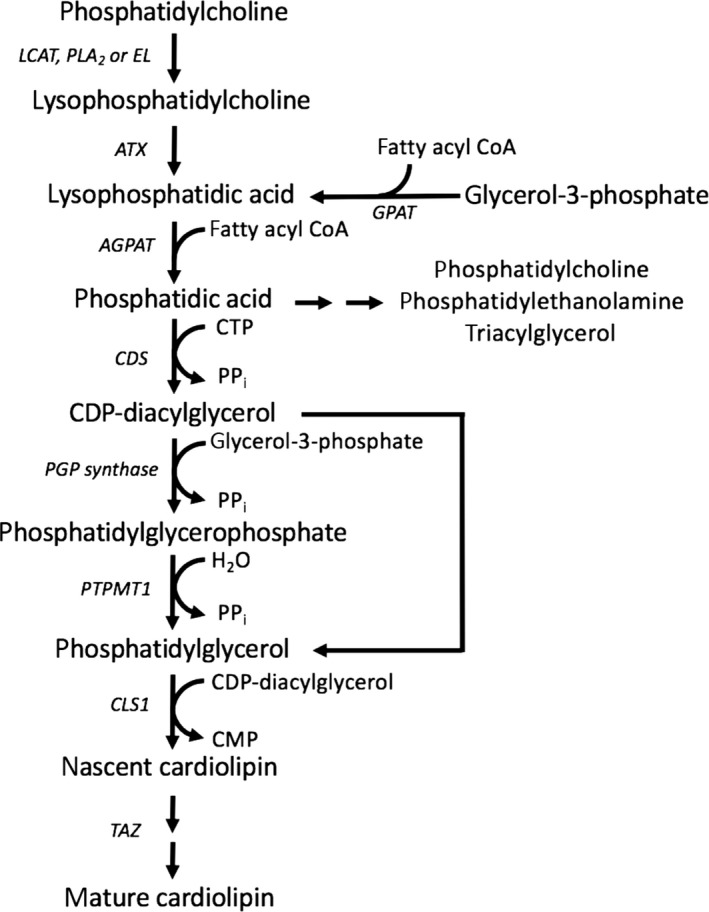
Synthesis pathway of cardiolipin from lysophosphatidylcholine and lysophosphatidic acid. AGPAT, acylglycerol‐3‐phosphate acyltransferase; ATX, autotaxin; CLS1, cardiolipin synthase; CDP, cytidine diphosphate; CDS, CDP‐diacylglycerol synthase; CMP, cytidine monophosphate; CTP, cytidine triphosphate; EL, endothelial lipase; GPAT, glycerol‐3‐phosphate acyltransferase; LCAT, phosphatidylcholine‐sterol acyltransferase; PTPMT1, phosphatidylglycerophosphate and protein‐tyrosine phosphatase 1; PGP synthase, phosphatidylglycerophosphate synthase; PLA_2_, phospholipase A_2_; PP_i_, pyrophosphate; TAZ, tafazzin

A potential biological mechanism by which LPC could influence mitochondrial oxidative capacity is through its role in the synthesis pathway of cardiolipin. Cardiolipin is a unique dimeric phospholipid containing four fatty acid chains that is specific to mitochondria. Cardiolipin is an essential constituent of mitochondrial membranes (Schlame & Greenberg, 2017). LPC in human plasma can be generated by the activity of: (a) phospholipase A_2_ (PLA_2_) on phosphatidylcholine; (b) by the activity of endothelial lipase (EL), including phospholipase A_1_ (PLA_1_), on high‐density lipoprotein (Gauster et al., 2005); (c) from phosphatidylcholine during the formation of cholesteryl esters (Figure 3). The synthesis of cardiolipin involves LPA and phosphatidic acid (PA). LPC can be hydrolyzed to LPA by autotaxin, a secreted glycoprotein that is widely expressed in tissues (Lyu et al., 2017; Perrakis & Moolenaar, 2014) (Figure 3). The membranes of both mitochondria and endoplasmic reticulum contain LPC (Pollard, Ortori, Stöger, Barrett, & Chakrabarti, 2017; Tsalouhidou et al., 2006; Veyrat‐Durebex et al., 2018). LPA can also be formed from the acylation of glycerol‐3‐phosphate on the outer mitochondrial membrane. Two isoforms of acylglycerol‐3‐phosphate acyltransferase (AGPAT 4 and 5) are located on the outer mitochondrial membrane and catalyze the acylation of LPA to PA (Gonzalez‐Baro & Coleman, 2017). PA is transferred to the inner mitochondrial membrane and is converted to nascent cardiolipin via CDP‐glycerol, phosphatidylglycerophosphate, and phosphatidylglycerol (Figure 3).

Nascent cardiolipin contains four fatty acid chains but then undergoes structural remodeling in which cardiolipin acquires a new set of fatty acids. The remodeling of cardiolipin is catalyzed by tafazzin, an enzyme that transfers fatty acids between phospholipids and lysophospholipids (Schlame & Greenberg, 2017). The dominant cardiolipin, comprising ~80%–90% of cardiolipin in mitochondria from normal human skeletal muscle in Westerners, is tetralinoleoyl cardiolipin (18:2)_4_ (Ikon & Ryan, 2017). Tafazzin does not show substrate preference and is not specific for linoleoyl chains; thus, the concept has emerged that fatty acid availability influences the final form of cardiolipin that is incorporated into mitochondrial inner membranes (Ikon & Ryan, 2017). The fatty acid species composition of cardiolipin may be critical. Cardiolipin influences membrane biophysical properties and shapes the curvature of the mitochondrial cristae, which affects the assembly and function of the electron transport chain complexes and generation of ATP (Paradies, Paradies, Benedictis, Ruggiero, & Petrosillo, 2014; Pennington et al., 2017).

Dietary intake of lipids can influence the fatty acid species composition of cardiolipin and mitochondrial bioenergetics (Monteiro, Oliveria et al., 2013a; Monteiro, Pereira et al., 2013). Significant differences in major species of cardiolipin and mitochondrial bioenergetics were found in an experimental rat model following feeding with a rapeseed oil‐based diet (Monteiro, Oliveria et al., 2013a; Monteiro, Pereira et al., 2013). Moreover, a DHA‐rich diet led to a high content of the tetra‐docosahexanoyl cardiolipin (22:6_4_) (Hussein et al., 2009). Higher dietary intake of linoleate (18:2) increased the incorporation of 18:2 in the fatty acid profile of cardiolipin in heart and brain mitochondria in rats (McGee, Lieberman, & Greenwood, 1996). Low plasma concentrations of certain LPC species, such as 18:1 and 18:2, could potentially impact the final fatty acid composition of cardiolipin. In humans, plasma LPC 18:2 concentrations generally decrease with age (Trabado et al., 2017).

Multiple machine‐learning algorithms with cross‐validated estimates showed that the six LPC species classified individuals in the highest quartile of *k*_PCr_ compared to those in the lower three quartiles. In previous studies involving *k*_PCr_ in the Baltimore Longitudinal Study of Aging, height was included as a covariate in multivariable analyses (Choi et al., 2016; Fabbri et al., 2017). In the present study, multivariable analyses showed that the relationship of LPC with *k*_PCr_ was more significant when height was included as a covariate. Further studies are needed to understand how anthropometric measures could influence the relationship of LPC with mitochondrial oxidative capacity.

The strengths of this study are the sample of well‐characterized community‐dwelling adults with a relatively low burden of disease, standardized collection of fasting plasma samples, mitochondrial oxidative capacity, and measurement of LPC using LC‐MS/MS. The limitations of this study include that the findings from this study cannot necessarily be generalized to other study populations. The BLSA is a convenience sample of adults, not a population‐based study. BLSA participants tend to be healthier, more educated, and have higher socioeconomic status than the general population. The BLSA may be unique in the collection of a large amount of data on skeletal muscle mitochondrial oxidative capacity in an ongoing cohort study of aging. Replication of the findings of the present study in another ongoing cohort is possible but would require substantial resources and time.

Future work is needed to characterize the relationship of low circulating LPC with decline of skeletal muscle oxidative capacity over time and to examine the relationship of circulating LPC with the fatty acid composition of cardiolipin in skeletal muscle.

## 4 EXPERIMENTAL PROCEDURES

### 4.1 Study subjects

The study subjects consisted of 385 participants, aged 25–97 years, in the Baltimore Longitudinal Study of Aging (BLSA) who had study visits between April 2013 and June 2016. The BLSA is a prospective open cohort study of community‐dwelling volunteers, largely from the Baltimore/Washington area. The study was established in 1958 and is described in detail elsewhere (Shock et al., 1984). Participants who are free of major chronic conditions and functional impairments are enrolled in the study and scheduled for follow‐up visits every 1 to 4 years, with more frequent follow‐up for older participants. The study herein was aimed at characterizing the cross‐sectional association of plasma LPC with mitochondrial oxidative capacity. Assessments were performed at an in‐patient study clinic and consisted of 2.5 days of medical, physiological, and psychological exams. The study protocol was approved by the institutional review boards of the National Institute of Environmental Health Science (NIH, North Carolina) and the Johns Hopkins School of Medicine Institutional Review Board. The study protocol was conducted in accordance with the 1964 Helsinki Declaration. At every visit, after the scope, procedures, and related risk were explained to participants, they consented to participate in the study and signed an informed consent document.

### 4.2 Mitochondrial oxidative capacity measured by magnetic resonance spectroscopy in vivo

Measurements of ^31^P‐MRS of phosphorus‐containing metabolites were obtained from the vastus lateralis muscle of the left thigh using a 3T Philips Achieva MRI scanner (Philips, Best, The Netherlands) and a 10‐cm ^31^P‐tuned flat surface coil (PulseTeq, Surrey, UK). Participants were placed in a supine position on the bed of the scanner, with a foam wedge placed under the knee to maintain slight flexion. The thighs and hips were secured with straps to minimize movement during exercise. After instruction and practice outside the MR magnet, participants performed rapid and intense ballistic knee extensions (Coen et al., 2013), achieving maximal recruitment of the quadriceps muscle. Depletion of phosphocreatine (PCr) was monitored throughout the exercise protocol, with exercise terminated after a 30%–70% reduction of the PCr signal height as compared with its value. ^31^P MRS spectral data were acquired throughout a baseline period of one minute immediately prior to the initiation of exercise, during exercise, and after exercise, using a pulse‐and‐collect sequence with adiabatic radiofrequency excitation pulses with a repetition time of 1.5 s. Four signal averages were performed for a time resolution of 6 s and total duration of MR data acquisition of 7.5 min (Choi et al., 2016). Spectra were processed using jMRUI (version 5.0), with metabolites quantified using a nonlinear least squares algorithm implemented through (AMARES) (Naressi, Couturier, Castang, Beer, & Graveron‐Demilly, 2001; Naressi, Couturier, Devos et al., 2001).

### 4.3 Skeletal muscle oxidative ATP resynthesis rate determined by ^31^P‐MRS

Postexercise PCr recovery rate was calculated by fitting the time‐dependent change in PCr peak area to the monoexponential recovery function: PCr (*t*) = PCr_0_ + ΔPCr(1‐*e* ^–^ *^t^* ^/PCr^), where PCr_0_ is the signal amplitude of phosphocreatine at the onset of PCr recovery after exercise, ΔPCr = (PCr_baseline_ – PCr_0_) is the decrease in PCr signal area during in‐magnet exercise from the baseline value PCr_baseline_ to the end‐exercise value PCr_0_, and _PCr_ is the PCr recovery time constant. The PCr recovery rate constant *k*_PCr_ = 1/_PCr_ is an index of in vivo oxidative capacity of skeletal muscle with no or minimal contribution from anaerobic metabolism. [PCr_baseline_], the intial concentration of PCr at rest, was estimated using a fully relaxed ^31^P MR spectrum through normalization of the PCr peak by ‐ATP, under the assumption that [ATP] = 5.5 mmol/kg weight at rest.

### 4.4 Collection of plasma

Blood was collected from participants who stayed overnight at the NIA Clinical Research Unit, Medstar Harbor Hospital in Baltimore, Maryland following a standardized protocol. Blood samples were drawn from the antecubital vein between 07:00 and 08:00 hr after an overnight fast. Participants were not allowed to smoke, engage in physical activity, or take medications before the blood sample was collected. Blood samples were immediately stored at 4°C, centrifuged within 4 hr, then immediately aliquoted and frozen at −80°C.

### 4.5 Measurement of plasma metabolites

Plasma metabolites were measured using LC‐MS/MS. Metabolites were extracted and concentrations are measured using the AbsoluteIDQ p180 kit (Biocrates Life Sciences AG, Innsbruck, Austria) following the manufacturer's protocol for a 5500 QTrap (Sciex, Framingham, MA, USA) mass spectrometer equipped with an electrospray ionization source, a Shimadzu CBM‐20A command module, LC‐20AB pump, and a Shimadzu SIL‐20AC‐HT autosampler, a CTO‐10Ac column oven heater, and running with Analyst 1.5.2 software. Briefly, 10 μL of plasma was pipetted onto a 96‐well Biocrates kit. The samples were dried for 30 min at room temperature (RT) under a stream of nitrogen. Fifty µL of 5% PITC reagent was added after which the reaction proceeded at RT for 20 min. The plate was then dried under nitrogen for 1 hr. Three hundred µL of 5 mM ammonium acetate in methanol was added and incubated at RT for 30 min on a shaker (450 rpm). The plate was centrifuged at 500 x g for 2 min and labeled; 50 µl of each sample was transferred to a 96‐deep‐well LC plate, and 10 µL of each sample was transferred to the 96‐deep‐well FIA plate. Four hundred fifty µL of 40% methanol in water was added to the LC plate. Four hundred ninety µL of FIA running solvent was added to the FIA plate. Ten µL was injected onto the Eclipse XDB C18, 3.5 μm, 3.0 × 100 mm with a Phenomenex C18 Security Guard Cartridge, 3.0 mm ID. The mobile phase consisted of solvent A (water containing 0.2% formic acid) and solvent B (acetonitrile containing 0.2% formic acid). The following gradient was used: 0–0.5 min 0% B, 5.5 min: 95% B; 6.5 min: 95% B; 7.0 min: 0% B; 9.5 min: 0% B. LC plate evaluation of the samples was carried out using the MetIDQ software. The FIA plate was run with 20 µl injection directly onto the MS at a flow of 30 µl/min with water/acetonitrile (1:1) containing 0.2% formic acid as the mobile phase. The following flow rate program was used: 0–1.6 min: 30 µl/min; 2.4 min: 200 µl/min; 2.80 min: 200 µl/min; and 3.00 min: 30 µl/min. Analyst/MetIDQ software was used to calculate concentrations in µmol/L. The total number of carbon atoms and double bonds in fatty acid chains in lysophosphatidylcholines is represented by “*x*:*y*,” where *x* denotes the number of carbons and y denotes the number of double bonds. The kit potentially measures 14 LPC, but LPC 14:0, 24:0, 26:0, 26:1, 28:0, and 28:1 were excluded from the analysis because they were below the limit of detection in nearly all subjects. The MS spectra were evaluated using Analyst/MetIDQ (Biocrates) software. Human plasma samples spiked with standard metabolites were used to monitor the reproducibility of the assay. The inter‐assay and intra‐assay coefficients of variation ranged from 5% to 15% for nearly all analytes.

### 4.6 Statistical analysis

Demographic and other characteristics were compared across quartiles of *k*_PCr_ using Wilcoxon rank‐sum test for continuous variables and Pearson chi‐square test for categorical variables. Multivariable linear regression models were used to examine the relationship between plasma lysophosphatidylcholines and the highest quartile versus the bottom three quartiles of *k*_PCr_. Classification modeling with eight machine‐learning algorithms was used to examine the ability of the six LPC to classify the highest quartile of mitochondrial oxidative capacity from the lower three quartiles. SuperLearner is an ensembling approach to machine learning is based upon the idea that no single machine‐learning algorithm is optimal for all data sets (van der Laan et al., 2007). The SuperLearner algorithm was used to fit, internally cross‐validate, and combine the results into a single classifier, as described in detail elsewhere; Semba et al., 2016). First, multiple individual machine‐learning classification algorithms (e.g., logistic regression) were implemented, using only a portion of the data to train each algorithm. Second, we combined estimates using the data left out of the training data, producing a cross‐validated estimate. The cross‐validated results from the individual machine‐learning algorithms were then combined via weighted average to minimize a cross‐validated mean squared error. The weighted average potentially fits the data better than each individual algorithm. Cross‐validated SuperLearner additionally leaves out a portion of data in the second step to produce cross‐validated estimates of the weights. SuperLearner tests multiple machine‐learning algorithms and optimally combines the results to find the lowest mean squared error (Polley & van der Laan, 2015; Rose, 2013). SuperLearner was applied by splitting the data into ten mutually exclusive and collectively exhaustive groups.

We selected eight individual machine‐learning algorithms to protect against model misspecification: (a) glmnet() in the glmnet package with alpha = 1 (least absolute shrinkage and selection operator [LASSO] regularization) (Friedman, Hastie, & Tibshirani, 2010), (b) glmnet() with alpha = 0 (ridge regression regularization) (Friedman et al., 2010), (c) glmnet() with alpha = 0.5 (elastic net regularization, which is a hybrid of LASSO and ridge regression regularization) (Friedman et al., 2010), (d) gbm() in the gbm package (generalized boosted regression) (Friedman, 2001; Friedman et al., 2010) with 10,000 trees and interaction depth = 2, (e) randomForest() in the randomForest package (random forest ensemble of classification and regression trees) with 1,000 trees (Breiman, 2001), (f) ipredbagg() in the ipred() package (bootstrap aggregation [BAGGing] of classification and regression trees) (Breiman, 2001) with 100 replicates, (g) earth() in the earth package (Milborrow, 2015) (an implementation of adaptive regression using piecewise linear splines) (Friedman 1991), and (h) bayesglm() in the arm package (main effects Bayesian logistic regression) with a Cauchy prior with scale = 2.5. Three algorithms had positive weights and contributed to the final classifier: ridge regression (Friedman et al., 2010), random forests (Breiman, 2001), and Bayes generalized linear models. Super Learner was carried out using the SuperLearner() and CV.SuperLearner() function in the SuperLearner package in R version 3.2.0.

## CONFLICT OF INTEREST

The authors declare no conflicts of interest.

## References

[acel12915-bib-0001] Aikawa, S. , Hashimoto, T. , Kano, K. , & Aoki, J. (2015). Lysophosphatidic acid as a lipid mediator with multiple biological actions. Journal of Biochemistry, 157, 81–89. 10.1093/jb/mvu077 25500504

[acel12915-bib-0002] Breiman, L. (2001). Random forests. Machine Learning, 45, 5–32.

[acel12915-bib-0003] Choi, S. , Reiter, D. A. , Shardell, M. , Simonsick, E. M. , Studenski, S. , Spencer, R. G. , … Ferrucci, L. (2016). ^31^P magnetic resonance spectroscopy assessment of muscle bioenergetics as a predictor of gait speed in the Baltimore Longitudinal Study of Aging. Journals of Gerontology. Series A, Biological Sciences and Medical Sciences, 71, 1638–1645. 10.1093/gerona/glw059 PMC5106855 27075894

[acel12915-bib-0004] Coen, P. M. , Jubrias, S. A. , Distefano, G. , Amati, F. , Mackey, D. C. , Glynn, N. W. , … Goodpaster, B. H. (2013). Skeletal muscle mitochondrial energetics are associated with maximal aerobic capacity and walking speed in older adults. Journals of Gerontology. Series A, Biological Sciences and Medical Sciences, 68, 447–455. 10.1093/gerona/gls196 PMC3593613 23051977

[acel12915-bib-0005] Fabbri, E. , Chia, C. W. , Spencer, R. G. , Fishbein, K. W. , Reiter, D. A. , Cameron, D. , … Ferrucci, L. (2017). Insulin resistance is associated with reduced mitochondrial oxidative capacity measured by ^31^P‐magnetic resonance spectroscopy in participants without diabetes from the Baltimore Longitudinal Study of Aging. Diabetes, 66, 170–176. 27737951 10.2337/db16-0754 PMC5204309

[acel12915-bib-0006] Friedman, J. H. (2001). Greedy function approximation: A gradient boosting machine. Annals of Statistics, 29, 1189–1232. 10.1214/aos/1013203451

[acel12915-bib-0007] Friedman, J. H. (1991). Multivariate adaptive regression splines (with discussion). Annals of Statistics, 19, 1–141.

[acel12915-bib-0008] Friedman, J. , Hastie, T. , & Tibshirani, R. (2010). Regularization paths for generalized linear models via coordinate descent. Journal of Statistical Software, 33, 1–22. 20808728 PMC2929880

[acel12915-bib-0009] Gauster, M. , Rechberger, G. , Sovic, A. , Hörl, G. , Steyrer, E. , Sattler, W. , & Frank, S. (2005). Endothelial lipase releases saturated and unsaturated fatty acids of high density lipoprotein phosphatidylcholine. Journal of Lipid Research, 46, 1517–1525. 10.1194/jlr.M500054-JLR200 15834125

[acel12915-bib-0010] Gonzalez‐Baro, M. R. , & Coleman, R. A. (2017). Mitochondrial acyltransferases and glycerophospholipid metabolism. Biochimica Et Biophysica Acta (BBA) ‐ Molecular and Cell Biology of Lipids, 1862, 49–55. 10.1016/j.bbalip.2016.06.023 27377347

[acel12915-bib-0011] Gonzalez‐Freire, M. , de Cabo, R. , Bernier, M. , Sollott, S. J. , Fabbri, E. , Navas, P. , & Ferrucci, L. (2015). Reconsidering the role of mitochondria in aging. Journals of Gerontology. Series A, Biological Sciences and Medical Sciences, 70, 1334–1342. 10.1093/gerona/glv070 PMC4612387 25995290

[acel12915-bib-0012] Hussein, N. , Federova, I. , Moriguchi, T. , Hamazaki, K. , Kim, H.‐Y. , Hoshiba, J. , & Salem, N. Jr (2009). Artificial rearing of infant mice leads to n‐3 fatty acid deficiency in cardiac, neural and peripheral tissues. Lipids, 44, 685–702. 10.1007/s11745-009-3318-2 19588181 PMC2771777

[acel12915-bib-0013] Ikon, N. , & Ryan, R. O. (2017). Cardiolipin and mitochondrial cristae organization. Biochim Biophys Acta Biomembranes, 1859, 1156–1163. 10.1016/j.bbamem.2017.03.013 28336315 PMC5426559

[acel12915-bib-0014] Kent, J. A. , & Fitzgerald, L. F. (2016). In vivo mitochondrial function in aging skeletal muscle: Capacity, flux, and patterns of use. Journal of Applied Physiology, 121, 996–1003. 27539499 10.1152/japplphysiol.00583.2016

[acel12915-bib-0015] López‐Otín, C. , Blasco, M. A. , Partridge, L. , Serrano, M. , & Kroemer, G. (2013). The hallmarks of aging. Cell, 153, 1194–1217. 10.1016/j.cell.2013.05.039 23746838 PMC3836174

[acel12915-bib-0016] Lyu, L. , Wang, B. , Xiong, C. , Zhang, X. , Zhang, X. , & Zhang, J. (2017). Selective export of autotaxin from the endoplasmic reticulum. Journal of Biological Chemistry, 292, 7011–7022. 10.1074/jbc.M116.774356 28298439 PMC5409469

[acel12915-bib-0017] Mapstone, M. , Cheema, A. K. , Fiandaca, M. S. , Zhong, X. , Mhyre, T. R. , MacArthur, L. H. , … Federoff, H. J. (2014). Plasma phospholipids identify antecedent memory impairment in older adults. Nature Medicine, 20, 415–418. 10.1038/nm.3466 PMC5360460 24608097

[acel12915-bib-0018] McGee, C. D. , Lieberman, P. , & Greenwood, C. E. (1996). Dietary fatty acid composition induces comparable changes in cardiolipin fatty acid profile of heart and brain mitochondria. Lipids, 31, 611–616. 10.1007/BF02523831 8784741

[acel12915-bib-0019] Milborrow, S. (2015). Earth: Multivariate adaptive regression spline models, Package version 4.4‐3. Vienna, Austria: R Foundation for Statistical Computing.

[acel12915-bib-0020] Monteiro, J. P. , Oliveira, P. J. , & Jurado, A. S. (2013a). Mitochondrial membrane lipid remodeling in pathophysiology: A new target for diet and therapeutic interventions. Progress in Lipid Research, 52, 513–528. 23827885 10.1016/j.plipres.2013.06.002

[acel12915-bib-0021] Monteiro, J. P. , Pereira, C. V. , Silva, A. M. , Maciel, E. , Baldeiras, I. , Peixoto, F. , … Oliveira, P. J. (2013). Rapeseed oil‐rich diet alters hepatic mitochondrial membrane lipid composition and disrupts bioenergetics. Archives of Toxicology, 87, 2151–2163. 23636270 10.1007/s00204-013-1068-7

[acel12915-bib-0022] Naressi, A. , Couturier, C. , Castang, I. , de Beer, R. , & Graveron‐Demilly, D. (2001). Java‐based graphical user interface for MRUI, a software package for quantitation of in vivo/medical magnetic resonance spectroscopy signals. Computers in Biology and Medicine, 31, 269–286. 10.1016/S0010-4825(01)00006-3 11334636

[acel12915-bib-0023] Naressi, A. , Couturier, C. , Devos, J. M. , Janssen, M. , Mangeat, C. , de Beer, R. , & Graveron‐Demilly, D. (2001). Java‐based graphical user interface for the MRUI quantitation package. MAGMA, 12, 141–152. 10.1007/BF02668096 11390270

[acel12915-bib-0024] Paradies, G. , Paradies, V. , De Benedictis, V. , Ruggiero, F. M. , & Petrosillo, G. (2014). Functional role of cardiolipin in mitochondrial bioenergetics. Biochimica et Biophysica Acta, 1837, 408–417. 24183692 10.1016/j.bbabio.2013.10.006

[acel12915-bib-0025] Pennington, E. R. , Fix, A. , Sullivan, E. M. , Brown, D. A. , Kennedy, A. , & Shaikh, S. R. (2017). Distinct membrane properties are differentially influenced by cardiolipin content and acyl chain composition in biomimetic membranes. Biochimica et Biophysica Acta, 1859, 257–267. 10.1016/j.bbamem.2016.11.012 27889304 PMC5217480

[acel12915-bib-0026] Perrakis, A. , & Moolenaar, W. H. (2014). Autotaxin: Structure‐function and signaling. Journal of Lipid Research, 55, 1010–1018. 10.1194/jlr.R046391 24548887 PMC4031933

[acel12915-bib-0027] Pollard, A. K. , Ortori, C. A. , Stöger, R. , Barrett, D. A. , & Chakrabarti, L. (2017). Mouse mitochondrial lipid composition is defined by age in brain and muscle. Aging (Albany NY), 9, 986–998. 10.18632/aging.101204 28325886 PMC5391243

[acel12915-bib-0028] Polley, E. C. , & van der Laan, M. J. (2015). SuperLearner: Super learner prediction, Package version 2.0‐15. Vienna, Austria: R Foundation for Statistical Computing.

[acel12915-bib-0029] Rose, S. (2013). Mortality risk score prediction in an elderly population using machine learning. American Journal of Epidemiology, 177, 443–452. 10.1093/aje/kws241 23364879

[acel12915-bib-0030] Schlame, M. , & Greenberg, M. L. (2017). Biosynthesis, remodeling and turnover of mitochondrial cardiolipin. Biochimica et Biophysica Acta, 1862, 3–7. 27556952 10.1016/j.bbalip.2016.08.010 PMC5125896

[acel12915-bib-0031] Semba, R. D. , Shardell, M. , Trehan, I. , Moaddel, R. , Maleta, K. M. , Ordiz, M. I. , … Manary, M. J. (2016). Metabolic alterations in children with environmental enteric dysfunction. Scientific Reports, 6, 28009. 27294788 10.1038/srep28009 PMC4904796

[acel12915-bib-0032] Shock, N. W. , Greulick, R. C. , Andres, R. , Arenberg, D. , Costa, P. , Lakatta, E. , & Tobin, J.D. (1984) Normal human aging: The Baltimore study of aging (NIH publication No.84‐2450).

[acel12915-bib-0033] Trabado, S. , Al‐Salameh, A. , Croixmarie, V. , Masson, P. , Corruble, E. , Fève, B. , … Chanson, P. (2017). The human plasma‐metabolome: Reference values in 800 French healthy volunteers; impact of cholesterol, gender and age. PLoS One, 12, e0173615 10.1371/journal.pone.0173615 28278231 PMC5344496

[acel12915-bib-0034] Tsalouhidou, S. , Argyrou, C. , Theofilidis, G. , Karaoglanidis, D. , Orfanidou, E. , Nikolaidis, M. G. , … Mougios, V. (2006). Mitochondrial phospholipids of rat skeletal muscle are less polyunsaturated than whole tissue phospholipids: Implications for protection against oxidative stress. Journal of Animal Science, 84, 2818–2825. 16971584 10.2527/jas.2006-031

[acel12915-bib-0035] van der Laan, M. J. , Polley, E. C. , & Hubbard, A. E. (2007). Super learner. Statistical Applications in Genetics and Molecular Biology, 6(1). 10.2202/1544-6115.1309 17910531

[acel12915-bib-0036] Veyrat‐Durebex, C. , Bocca, C. , Chupin, S. , Kouassi Nzoughet, J. , Simard, G. , Lenaers, G. , … Blasco, H. (2018). Metabolomics and lipidomics profiling of a combined mitochondrial plus endoplasmic reticulum fraction of human fibroblasts: A robust tool for clinical studies. Journal of Proteome Research, 17, 745–750. 10.1021/acs.jproteome.7b00637 29111762

[acel12915-bib-0037] Ward‐Caviness, C. K. , Xu, T. , Aspelund, T. , Thorand, B. , Montrone, C. , Meisinger, C. , … Peters, A. (2017). Improvement of myocardial infarction risk prediction via inflammation‐associated metabolite biomarkers. Heart, 103, 1278–1285. 10.1136/heartjnl-2016-310789 28255100 PMC5871235

